# Influence of Sleeping Patterns in Health and Academic Performance Among University Students

**DOI:** 10.3390/ijerph17082760

**Published:** 2020-04-16

**Authors:** María Dolores Toscano-Hermoso, Félix Arbinaga, Eduardo J. Fernández-Ozcorta, Juan Gómez-Salgado, Carlos Ruiz-Frutos

**Affiliations:** 1 Faculty of Nursing, Department of Nursing, University of Huelva, 21007 Huelva, Spain; mdolores.toscano@denf.uhu.es; 2 Faculty of Education, Psychology and Sports Science, Department of Clinical and Experimental Psychology, University of Huelva, 21007 Huelva, Spain; 3 Department of Physical Activity and Sports, Center for University Studies Cardenal Spínola CEU, University of Seville attached centre, 41930 Bormujos, Spain; ejfernandez@ceuandalucia.es; 4 School of Labour Sciences, Department of Sociology, Social Work and Public Health, University of Huelva, 21007 Huelva, Spain; frutos@uhu.es; 5 Safety and Health Posgrade Program, University Espiritu Santo, 092301 Guayaquil, Ecuador

**Keywords:** chronotype, gender, individual differences, lifestyle, nightmares, sleep quality, university students

## Abstract

Sleep problems in university students are important and have implications for health, quality of life, and academic performance. Using an ex post facto design, a total sample of 855 students (55.7% women) participated in the study. Sleep assessment was conducted using the Pittsburgh Sleep Quality Index, the Nightmare Frequency Scale, the Nightmare Proneness Scale, and the Composite Morningness Scale. Women show a higher risk [OR = 2.61] of presenting poor sleep quality (> 5 points on the PSQI) compared with men (*p* < 0.001). Similarly, women reported a greater frequency of nightmares (*p* < 0.001, *d* = 0.60), greater propensity for nightmares (*p* < 0.001, *d* = 0.70) and a higher score on Item-5h of the PSQI regarding nightmares (*p* < 0.001, *d* = 0.59). Women, compared with men, show higher risk [OR = 2.84] for a sleep disorder related to nightmares (*p* = 0.012). Women need more time to reach a state of alertness after getting up (*p* = 0.022), and there was an interaction between sex and the alertness factor when evaluating the subjective quality of sleep (*p* = 0.030). Women show worse sleep quality and a higher frequency and propensity for suffering nightmares. When considering the relationship between sleep quality and academic performance, it is observed that students with poor sleep quality obtain lower academic scores (M = 7.21, SD = 0.805) than those with good sleep quality (M = 7.32, SD = 0.685), an effect that reaches significance (*t* = 2.116, *p* = 0.035). Regarding the relationship between the categorized chronotype and academic performance, students with a morning chronotype achieve better academic results (M = 7.41, SD = 0.89) than their evening counterparts (M = 7.15, SD = 0.76), although these differences have a small effect size (*d* = 0.31).

## 1. Introduction

Sleep serves a fundamental physiological function for human beings, and the quantity and quality of required sleep depends on a number of interacting environmental factors and underlying physiological variables [1,2,3,4,5].

Sleep plays an important role in learning and memory formation. Sleeping facilitates the process of memory consolidation, as it provides the optimal conditions for effective reorganization of representations [6]. According to Rauchs et al. sleep promotes the consolidation of both spatial and contextual memories [7]. During sleep, neurophysiological processes occur which forward the integrations of new data into pre-existing cortical networks [8]. This electrophysiological hippocampal activity has been associated with memory consolidation [9] and learning [10].

Sleep problems have been linked to learning difficulties, neurocognitive performance, and poor academic performance [11,12,13]. Previous studies have reported that more than 60% of the university population show sleep problems [14] and more than 25% sleep < 7 h/night, as measured by indices reflecting poor sleep quality [15]. Between 16–23% of university students report insomnia symptoms [16,17,18] and significant mental health problems related to sleep disturbances [19]. 

Problems related to sleep differ according to sex and age throughout the life cycle [20,21]. Some authors have identified several socio-demographic variables, risk behaviors, and factors related to the duration of sleep and associated health states [15,22]. Among other factors associated with sleep problems, gastrointestinal diseases are indicated [23,24,25,26], along with nightmares [27] and chronotype [28].

Recent studies suggest the existence of a strong association between sleep disorders and gastrointestinal diseases [23,25,29]. In this regard, it has been found that women suffer from a greater number of gastrointestinal disorders, which are imbalances that are linked to biological issues derived from and related to the nervous system [30] and endocrine system [31].

Nightmares are defined as vivid dreams that are associated with intense negative emotions, which can cause the person to awaken from sleep [32]. Nightmares are related to a wide variety of clinical problems and disorders [33,34,35,36,37] and can be regarded as constituting a specific disorder [32]. 

It is estimated that 45% of the general adult population has suffered nightmares during the last month [38] and around 2–5% suffer them at least once a week [37,39]. In the adolescent and adult population there are gender differences in the prevalence of nightmares, with women reporting to suffer a greater number of nightmares than men [20,40].

With respect to chronotype, no consensus has been reached in terms of establishing gender differences. Whilst it has been reported that women obtain higher scores on morningness [41,42], other studies have indicated that the morning cycle is more frequent in men and the evening pattern more frequent in women [43]. It is indicated that the chronotypic influence, together with a probable gender difference in the neurophysiological substratum of emotional processing, can give rise to the differential appearance of nightmares for women with the eveningness chronotype [44].

Further, university students are associated with a preference for eveningness, associated with lower academic performance [45]. A recent meta-analysis of circadian preference and academic achievement showed that evening-orientated students were related to worse academic performance [46]. According to Zerbini et al., this effect is mediated by conscientiousness, learning/achieving motivation, mood, and alertness [47].

In this context, the objective is to analyze, according to the gender of university students, possible differential patterns related to sleep. This study contributes to filling the gap in knowledge on the influence of students’ gender on sleeping patterns. In particular, we expected to find that women, in comparison with men, present a greater frequency and propensity for nightmares, as well as poorer subjective sleep quality. Moreover, we expect to find no differences between men and women in terms of the chronotype, although women who report having digestive disease are expected to show greater problems with sleep. Therefore, we expect to confirm the existing evidence that women of this age, compared with men, show a greater number of problems with sleep and a higher score on subjective quality of sleep, which is indicative of poorer quality. Finally, we set out to confirm whether those students with sleep problems are more likely to show impaired academic performance.

## 2. Materials and Methods 

### 2.1. Design 

Using an ex post facto design, an incidental sampling procedure was used to select students from various University Degree courses. All the participants completed the informed consent form and the tests were administered in a designated classroom during a period in which there were no exams. The study was approved by the university’s bioethics committee and followed the recommendations of the Declaration of Helsinki (1975–2000). 

### 2.2. Sample 

A total sample of 855 university students participated in the study (476 women (55.7%) and 378 men (44.3%)), with an average age of 22.55 years (SD = 4.851). Through an ad hoc interview information was gathered on: sex, age, average academic score (minimum: 0 and maximum: 10), subjective assessment of academic achievement (from 0: Very bad to 10: Very good) and the existence of disease (0: none, 1: muscular, 2: bone, 3: respiratory, 4: cardiovascular, 5: digestive, 6: other).

### 2.3. Assessment Tools

We assessed the participants’ subjective perception of their sleep quality using the Pittsburgh Sleep Quality Index (PSQI) [48] in the version adapted to Spanish [49], with its high internal consistency (Cronbach’s alpha of 0.81). Buysse et al. also demonstrated the predictive validity of this instrument with a cut-off point of a PSQI score > 5 showing 89.6% sensitivity and 86.5% specificity for identifying poor sleep quality [48]. The 19 items within this instrument pertain to different determinants of sleep quality, and are grouped into seven components: (a) subjective sleep quality, (b) sleep latency, (c) sleep duration, (d) sleep efficiency, (e) sleep disturbances, (f) use of sleeping medications, and (g) daily dysfunction due to sleep (drowsiness, etc.). Responses to the PSQI yield seven component scores from 0 to 3, and the total PSQI score ranges from 0 to 21 points, with higher scores suggestive of poorer sleep quality. In this work, the internal consistency of the test was acceptable (*α* = 0.771).

To assess Nightmare Frequency, participants estimated the number of nightmares they experienced as measured by the Schredl Nightmare Frequency Scale [50]. Response options were 0 = never, 1 = less than once a year, 2 = about once a year, 3 = about 2 to 4 times a year, 4 = about once a month, 5 = about 2 to 3 times a month, 6 = about once a week, 7 = several times a week. The test-retest reliability of this single-item scale has been estimated at 0.75 across four weeks [51]. The measure has been correlated with hypothetically related variables, thus providing some evidence of construct validity [45]. Using the International Classification of Sleep Disorders, 2nd edition criteria [52], individuals who reported more than one nightmare per week (Option 7, “several times a week”) formed a group with a high probability of presenting sleep disorders related to nightmares.

The evaluation of nightmare proneness was conducted using the Nightmare Proneness Scale (NPS) [53]. The NPS includes 14 items; participants responded using a 7-point scale where 1 = “Strongly Disagree” and 7 = “Strongly Agree.” Responses were summed to produce a total NPS score, with higher scores indicating greater proneness to nightmares. The alpha coefficient of reliability for the 14 items was 0.88, and the test-retest correlation was 0.72 [53]. In the translation carried out for this work, which employed the back-translation method, the internal consistency obtained, as measured by the Cronbach alpha coefficient, was *α* = 0.848.

The chronotype was evaluated using the Composite Scale of Morningness (CSM) [36], in its Spanish version [54,55]. This instrument consists of 13 questions (4- or 5-point scale) about the time individuals wake up and go to bed, preferred times for physical and mental activity, and subjective alertness. This yields a total score (CSM-Total), a general morning factor (CSM-General) and an alertness factor (CSM-Alert).The morningness score is obtained by adding up the item scores, and ranges from 13 (eveningness) to 55 (morningness). The criteria to establish the type of chronotype are adopted as follows: evening (scores below the 10th percentile) and morning (scores above the 90th percentile) [54].

The reliability and validity of the CSM has been confirmed in previous studies using Spanish samples, with a Cronbach’s alpha of *α* = 0.85 [54,56]. In this work the reliability obtained using Cronbach’s alpha was quite acceptable for the CSM-Total (*α* = 0.807), for the CSM-General (*α* = 0.775), and for the CSM-Alert (*α* = 0.715).

### 2.4. Data Analysis

Descriptive analyzes of the different variables were carried out (M, SD). For comparisons between categorical variables, the *χ*2 statistic (*df*, *n*) and the corresponding effect size (Phi, Cramer’s V) were used. The Odds Ratio [OR] and the Student *t*-test were used to analyze the quantitative variables, along with their effect sizes (Cohen’s d) (small effect size: 0.2–0.3, medium effect size: around 0.5, and large effect size: > 08). Different bivariate correlations were calculated to verify the relationship between the total score of the PSQI, the propensity for nightmares, and the frequency of nightmares. A univariate analysis was conducted according to a general linear model to determine the relationship between gender and the effect of quality of sleep on both the average academic score and the subjective assessment of academic achievement. All statistical analyses were carried out using the SPSS 20 program (IBM, Armonk, NY, USA). 

## 3. Results

The men in our sample were older than the women, although the effect size is small. A greater number of women reported suffering from some disease (*χ*2(6,853) = 18.863, *p* = 0.004). When grouping diseases as "digestive or other diseases", it was found that women, compared with men, have a higher risk [OR = 4.76] of suffering from digestive diseases (*χ*2(1,855) = 9.840, *p* = 0.002, Phi = 0.107, 95% CI [1.632–13.885]). In terms of academic performance and the participants’ subjective assessment of such performance, there were no observable differences between the male and female students (Table 1).

When analyzing the subjective quality of sleep, as assessed by the PSQI, it was found that 56.6% of the sample showed poor sleep quality (PSQI > 5 points), of which 65.7% are women. Women showed an increased risk [OR = 2.61] of presenting poor sleep quality (PSQI scores > 5) compared with men, (*χ*2(1,854) = 45.606, *p* < 0.001, Phi = 0.233; 95% CI [1.97–3.46]). On the other hand, women (M = 7.82, SD = 1.28) reported spending the same number of hours in bed as men (M = 7.86, SD = 1.21), with a *t* = 0.480 (*p* = 0.631). However, women (M = 6.74, SD = 1.18) reported sleeping fewer hours than men (M = 7.03, SD = 1.07), with a *t* = 3.799 (*p* < 0.001). Consequently, sleep efficiency is lower in women (M = 86.77, SD = 11.39) compared with men (M = 90.12, SD = 9.11), with a *t* = 4.657 (*p* < 0.001).

Regarding the presence of diseases, those who claim to have digestive diseases show an increased risk [OR = 4.58] of experiencing poor sleep quality (PSQI > 5 points) compared with the group that report not having digestive disease (*χ*2(1,839) = 9.271, *p* = 0.002, Phi = 0.105, 95% CI [1.57–13.362]). By exploring the possible interaction between the presence of digestive diseases and gender on the quality of sleep and after performing a univariate analysis according to a general linear model, no interaction was found between these variables (F(1, 838) = 0.010, *p* = 0.922).

When considering the relationship between sleep quality and academic performance, it is observed that students with poor sleep quality (PSQI > 5 points) obtain a lower academic score (M = 7.21, SD = 0.805) than those with good quality of sleep (M = 7.32, SD = 0.685) an effect that reaches significance (*t* = 2.116, *p* = 0.035). After carrying out the univariate analysis it was possible to confirm that there is no interaction between gender and PSQI scores on academic performance (F(1, 827) = 1.111, *p* = 0.292).

Further, in terms of the subjective assessment of academic achievement achieved, it is also shown that those students with poor sleep quality show a lower assessment of attainment (M = 6.97, SD = 1.293) compared with those who report a good quality of sleep (M = 7.30, SD = 1.167) with a *t* = 3.817 (*p* < 0.001). There is no interaction between gender and PSQI scores (F(1, 827) = 0.105, *p* = 0.746) for the subjective assessment of academic achievement.

Table 2 displays the scores categorized on the various PSQI subscales and the risk associated with women. The results show the existence of differences according to gender, in which women show a higher risk of obtaining indicators of poor sleep quality on all the subscales of the test.

When evaluating nightmares, women report a higher score on the scale of nightmare propensity, on the frequency scale of nightmares, and on item 5h of the PSQI (Table 3). On the other hand, 3.6% of participants have a sleep disorder related to nightmares, according to the international classification of sleep disorders. In this regard, the female students show an increased risk [OR = 2.84] of presenting a sleep disorder related to nightmares compared with the male students (*χ*2(1,850) = 6.243, *p* = 0.012, Phi = 0.09; 95% CI [1.210–6.664]).

When the participants are grouped according to whether or not they report having digestive diseases, there are no differences in the frequency of nightmares (*t* = 1.537, *p* = 0.125) or in the score obtained on item 5h of the PSQI (*t* = 1288, *p* = 0.198). However, on the scale of propensity for nightmares the scores are higher in those who report having digestive diseases (M = 48.70, SD = 13.044) compared to the remainder of students (M = 36.61, SD = 12.778) with a *t* = 4.836 (*p* < 0.001). There were no interactions between gender and digestive disease for the propensity for nightmares (F(1,843) = 0.346, *p* = 0.557).

When analyzing the scores on the subscales of morningness (Table 4), women obtain significantly lower scores only on the CSM-Alert. The CSM-Alert shows negative correlations with the frequency of nightmares (*r* = −0. 135, *p* < 0.001), with Item 5h of the PSQI (*r* = −0.139, *p* < 0.001), with the propensity for nightmares (*r* = −0.255, *p* < 0.001) and with the total score on the PSQI (*r* = −0.281, *p* < 0.001). In this regard, gender has shown a significant interaction with the CSM-Alert score when assessing the subjective quality of sleep through the total score on the PSQI (F(9,839) = 2.065, *p* = 0.030), but there is no such interaction when the frequency of nightmares (F(9,849) = 0.774, *p* = 0.640), the propensity for nightmares (F(9, 843) = 0.752, *p* = 0.661) or Item 5h of the PSQI (F(9, 851) = 1,127, *p* = 0.340) are assessed.

Finally, when analyzing the relationship between the categorized chronotype (evening: < percentile 10 [*n* = 92 (10.8%)], morning: > 90th percentile [*n* = 102 (11.9%)]) no differences were observed according to the gender of the participants (*χ*2(1,194) = 1.395, *p* = 0.238), although there were differences in terms of sleep quality, where evening people reported to having poorer sleep quality (M = 7.79, SD = 3.459) compared with morning people (M = 5.75, SD = 3.764) with a *t* = 3.893 (*p* < 0.001). Thus, those with poor sleep quality show an increased risk [OR = 2.69] of being an ‘evening person’ compared with those who obtain a good quality of sleep, with a *χ*2(1.192) = 10.943, *p* = 0.001, Phi = 0.239; 95% CI [1,487–4,867].

Regarding the relationship between the categorized chronotype and academic performance, differences are observed (*t* = 2.127, *p* = 0.035). In particular, students with a morning chronotype achieve better academic results (M = 7.41, SD = 0.89) than their evening counterparts (M = 7.15, SD = 0.76), although these differences have a small effect size (*d* = 0.31).

## 4. Discussion

In the present work we attempted to analyze, according to the students’ gender, the differences in patterns related to the subjective quality of sleep, the frequency and propensity for nightmares, the existence of digestive disease, and the chronotype of the individual. The results confirmed our working hypotheses since women present a greater number of problems with sleep, as well as a score on the tests of subjective quality of sleep that are indicative of poor quality.

In relation to sleep patterns, the results indicate that in our sample the women reported sleeping fewer hours than men, which is different to what has been observed in the adult population [21,57]. However, the average number of hours of sleep found in our university students coincides with the average of the university population of other countries [14,22].

The data obtained on the percentage of students with poor sleep quality support the results of Lund et al. [14], who reported that approximately 60% of students have poor sleep quality, with women being those at greater risk. Thus, women report spending the same number of hours in bed as men, but they admit to sleeping fewer hours, thus having a lower sleep efficiency. All of these findings are compatible with those provided by previous studies, where women are more likely than men to report more sleep problems and poor sleep quality [58,59]. Similarly, other studies [60] report that, for a similar sleep schedule in men and women, women’s chronobiological cycle makes them sleep and wake up before men.

Analyzing the subscales of the PSQI, our results are similar to those of Tang et al. [21] who found significant differences in favor of men in terms of subjective sleep quality, sleep latency, duration of sleep, efficiency of sleep, and in sleep disturbances; although they did not find gender differences in the use of sleep medications. However, our data on diurnal dysfunctions are not compatible with those of Tang et al. [21], since in the present investigation there are significant differences in this component. These results differ from those found in young people, where no differences were found according to gender in the subjective quality of sleep, latency, duration and efficiency of sleep, as well as in sleep disturbances and diurnal dysfunction [42,61].

In line with the findings of several recent investigations [23,24,26], a relationship was found between the presence of digestive disease and the quality of sleep. Chen et al. indicated that reflux symptoms are a risk factor for poor sleep quality [24] and Ali reported an interdependent relationship between sleep quality and gastrointestinal diseases [23]. In this regard, it has been shown that poor sleep causes the exacerbation of gastrointestinal symptoms and that many gastrointestinal diseases affect the sleep-wake cycle, causing poor sleep [25]. Whilst in the present work it has been possible to confirm the relationship between digestive diseases and sleep problems, no differences have been found in terms of gender.

Regarding academic performance obtained by university students and its relationship with the quality of sleep, it has been found that sleep disorders correlate with difficulties in achieving academic success [11]. Moreover, the results for nightmares appear to agree with the majority of other existing investigations. Women report suffering from nightmares more frequently than men [20,62,63,64,65]. The data indicate that 3.6% of university students report having nightmares several times a week, a percentage lower than those previously reported [64]. Regarding the propensity or tendency to suffer from nightmares, no previous studies have been found that allow for distinguishing the data according to gender; however, the present work has shown that it is women who report a greater tendency to suffer from nightmares.

Chronotype analysis is one of the sleep-associated phenomena that has shown the greatest discrepancy in results. Indeed, on the basis of the reviews carried out, the literature appears to show contradictory data in this regard. Thus, whilst it has been possible to observe a higher prevalence of eveningness chronotype in males, both in samples of university students [41,42] and in the adult population [43], data has also been found to indicate a greater incidence of this chronotype in females with samples from the general population [66]. In our study, the data obtained do not indicate differences in terms of gender in the sample of students, a finding that is in accord with previous studies using adult samples from the general population [67]. On the other hand, the percentage of students classified as having eveningness chronotypes is lower than in previous studies using similar samples [68]. It has been found that university students with a morning chronotype show better academic results and those with an evening style chronotype show a poor quality of sleep [45,66,68].

The limitations of the present study include the type of design used, which does not allow for establishing causal relationships, along with the incidental nature of the sample. In future investigations, it would be useful to obtain representative groups of university students through randomized and stratified sampling and to apply experimental designs to determine the causality between the variables studied. It would also be of interest to complement the sleep evaluations with objective methods (such as actigraphy or polysomnography), although these limitations do not diminish the relevance and importance of the findings found through the use of questionnaires.

Finally, due to the strong influence of sleep on the academic performance and health of university students [17,45,58,69,70], it would be pertinent to develop preventive and educational initiatives aimed at optimizing sleep habits. It should be noted that a significant number of factors related to sleep problems are modifiable, and not only by pharmacological therapy; it has been confirmed, for instance, that a single one-hour session on education in sleep hygiene improves both the quantity and quality of sleep [71,72].

## 5. Conclusions

In conclusion, the data support the initial hypothesis that women have poorer quality of sleep compared with men. This is confirmed through the different indicators studied: sleep patterns, subjective quality of sleep, and in all the subscales (subjective quality, latency, duration, efficiency, sleep disturbances and diurnal dysfunctions) as well as frequency and propensity for suffering nightmares, whilst no differences were observed in terms of chronotype.

## Figures and Tables

**Table 1:** Descriptive characteristics of the simple of university students.

		Gender				
M (SD)	Tota l855	Male 379 (44.3%)	Female 476 (55.7%)	*t*	*p*	Cohen’s d
Age	22.55 (4.851)	22.97 (4.993)	22.21 (4.713)	2.262	0.024	0.16
Academic grade	7.25 (0.769)	7.30 (0.774)	7.22 (0.763)	1.601	0.110	
Achievement level evaluation	7.11 (1.264)	7.19 (1.247)	7.04 (1.274)	1.706	0.088	
Illness N (%)			*χ^2^_(6, 853)_* = 18.863 *p* = 0.004		*Cramer´s V* = 0.15	
No illness	695 (81.5)	330 (47.5)	365 (52.5)			
Muscular	12 (1.4)	4 (33.3)	8 (66.7)			
Osseous	21 (2.5)	7 (33.3)	14 (66.7)			
Respiratory	38 (4.5)	14 (36.8)	24 (63.2)			
Cardiovascular	5 (0.6)	2 (40)	3 (60)			
Digestive	27 (3.2)	4 (14.8)	23 (85.2)			
Other	55 (6.4)	17 (30.9)	38 (69.1)			

**Table 2:** Comparison of the scores of the categorised subscales of the Pittsburgh Sleep Quality Index (PSQI) and the Odds Ratio (OR) for women university students.

		Gender						
PSQI *N (%)*	Total (855)	Male (378, 44.3%)	Female (476, 55.7%)	*χ*2(1,854)	*p*	Phi	OR	95% CI
Component 1. Subjective quality^.a^				24.461	< 0.001	0.17	2.12	1.57–2.86
Poor	277 (32.4)	89 (32.1)	189 (67.9)					
Good	577 (67.6)	289 (50.1)	288 (49.9)					
Component 2. ^b^ Sleep latency				10.897	0.001	0.11	1.58	1.20–2.09
High	381 (44.8)	145 (38.1)	236 (61.9)					
Low	470 (55.2)	232 (49.4)	238 (50.6)					
Component 3. Sleep duration				12.332	< 0.001	0.12	2.12	1.38–3.24
< 7 hours	116 (13.6)	34 (29.3)	82 (70.7)					
> 7 hours	736 (86.4)	344 (46.7)	392 (53.3)					
Component 4. Sleep efficiency				12.670	< 0.001	0.12	2.29	1.44–3.65
< 75%	98 (11.5)	27 (27.6)	71 (72.4)					
> 75%	752 (88.5)	350 (46.5)	402 (53.5)					
Component 5. ^c^ Sleep alterations				15.708	< 0.001	0.14	2.15	1.47–3.17
High	146 (17.2)	43 (29.5)	103 (70.5)					
Low	701 (82.8)	332 (47.4)	369 (52.6)					
Component 6. Drug use				4.919	0.027	0.08	1.82	1.06–3.10
< 1 time/week	787 (92.2)	357 (45.4)	430 (54.6)					
> 1 time/Week	67 (7.8)	21 (31.3)	46 (68.7)					
Component 7. Daytime sleep dysfunctions ^d^				26.887	< 0.001	0.19	2.21	1.63–2.98
High	275 (32.3)	87 (31.6)	188 (68.4)					
Low	576 (67.7)	291 (50.5)	285 (49.5)					

*a.*- Poor (categories PSQI.- Very poor, Pretty poor), Good (categories PSQI.- Very good, pretty good); *b*.- High (categories PSQI.- 31–60 minutes, > 60 minutes), Low (categories PSQI.- <15 minutes, 16–30 minutes); *c*.- Low (categories PSQI.- scores between 0 y 1–9 points), High (categories PSQI.- scores between 10–18 y 19–27 points); *d*.- High (categories PSQI.- 1–2 times/week, > 3 times/week,) Low (categories PSQI.- < 1 time per week, Never in the last month).

**Table 3:** Comparison of the scores in the evaluation of nightmares depending on students’ gender.

		Gender				
M (SD)	Total (855)	Male (378, 44.3%)	Female (476, 55.7%)	*t*	*p*	Cohen’s d
Nightmare frequency *	3.50 (1.60)	2.98 (1.58)	3.91 (1.50)	8.692	< 0.001	0.60
Nightmare tendency **	36.99 (12.95)	32.26 (11.87)	40.77 (12.55)	10.023	< 0.001	0.70
Item 5h PSQI ***	0.70 (0.79)	0.45 (0.66)	0.89 (0.82)	8.578	< 0.001	0.59

* Nightmare frequency scale [45]. ** Nightmare tendency scale [48]. *** Frequency of “having nightmares or bad dreams in the last month” from the PSQI questionnaire PSQI [43].

**Table 4:** Comparison of the scores related to chronotype depending on students´ gender.

		Gender				
*M (SD)*	Total (855)	Male (378, 44.3%)	Female (476, 55.7%)	*t*	*p*	*Cohen’s d*
CSM-Total	32.44 (6.26)	32.80 (6.38)	32.25 (6.16)	1.501	0.134	
CSM-General	24.57 (5.06)	24.74 (5.11)	24.43 (5.02)	0.896	0.371	
CSM-Alert	7.87 (2.08)	8.05 (2.14)	7.72 (2.02)	2.291	0.022	0.16

CSM. Composite Scale of Morningness [49].
